# Association of uteroglobin-related protein 1 with smoke inhalation injury severity

**DOI:** 10.5935/0103-507X.20210035

**Published:** 2021

**Authors:** Sabrina Frighetto Henrich, Tatiana Helena Rech, Cristiane Ritter, Monique Michels, Felipe Dal-Pizzol, Gilberto Friedman

**Affiliations:** 1 Postgraduate Program in Pneumological Sciences, Universidade Federal do Rio Grande do Sul - Porto Alegre (RS), Brazil.; 2 Hospital de Clínicas de Porto Alegre, Universidade Federal do Rio Grande do Sul - Porto Alegre (RS), Brazil.; 3 Postgraduate Program in Medical Sciences: Endocrinology, Universidade Federal do Rio Grande do Sul - Porto Alegre (RS), Brazil.; 4 Postgraduate Program in Medical Sciences, Universidade do Extremo Sul Catarinense - Criciúma (SC), Brazil.

**Keywords:** Uteroglobin, Bronchoscopy, Burns, Smoke inhalation injury, Morbidity, Mortality, Uteroglobulina, Broncoscopia, Queimaduras, Lesão por inalação de fumaça, Morbidade, Mortalidade

## Abstract

**Objective:**

To evaluate serum uteroglobin-related protein 1 expression early after smoke inhalation injuries and its association with the severity of inhalation injury in burned patients.

**Methods:**

Smoke or chemical inhalation injury is associated with morbidity and mortality. The consequences of inhalation result from an inflammatory response. Uteroglobin-related protein 1 is an anti-inflammatory protein and may improve lung inflammation. We hypothesized that uteroglobin-related protein 1 levels could reflect disease severity and predict outcome in patients with inhalation injury. Sixteen patients diagnosed with acute respiratory distress syndrome secondary to smoke inhalation injury were prospectively included in the study. Plasma was collected upon intensive care unit admission and within 24 hours of the inhalation injury. Bronchoscopies were carried out in all patients to assess the severity of inhalation injury within 72 hours. Uteroglobin-related protein 1 plasma levels were determined in duplicate with enzyme-linked immunosorbent assay.

**Results:**

The mean age was 23 ± 5 years, and the inhalation injury distribution was as follows: three of grade 1, four of grade 2, and nine of grade 3. The level of uteroglobin-related protein 1 was related to inhalation severity (grade 1: 0.389 ± 0.053 arbitrary units *versus* grade 2: 0.474 ± 0.0423 arbitrary units *versus* grade 3: 0.580 ± 0.094 arbitrary units; p = 0.007).

**Conclusion:**

Plasma levels of uteroglobin-related protein 1 are associated with the degree of lung inhalation injury.

## Abstract (pt)

**Objetivo:**

Avaliar a expressão sérica da proteína 1 relacionada à uteroglobulina na fase inicial após lesões por inalação de fumaça e sua associação com a gravidade da lesão por inalação em pacientes queimados.

**Métodos:**

A lesão por inalação de fumaça ou produtos químicos se associa com morbidade e mortalidade. As consequências da inalação resultam de uma resposta inflamatória. A proteína 1 relacionada à uteroglobulina é anti-inflamatória e pode melhorar a inflamação pulmonar. Nossa hipótese é que os níveis de proteína 1 relacionada à uteroglobulina podem refletir a gravidade da doença e predizer o desfecho em pacientes com lesão por inalação. Incluíram-se prospectivamente neste estudo 16 pacientes com diagnóstico de síndrome do desconforto respiratório agudo decorrente de lesão por inalação de fumaça. Em todos os pacientes, colheu-se amostra de plasma quando da admissão à unidade de terapia intensiva, para avaliar a gravidade da lesão por inalação dentro de 72 horas. Os níveis plasmáticos de proteína 1 relacionada à uteroglobulina foram determinados em duplicata por meio de ensaio de imunoabsorção ligado à enzima.

**Resultados:**

A média de idade foi de 23 ± 5 anos, e a distribuição da lesão por inalação foi: três em grau 1, quatro em grau 2 e nove em grau 3. O nível de proteína 1 relacionada à uteroglobulina foi relacionado ao grau de severidade (grau 1: 0,389 ± 0,053 unidade arbitrária versus grau 2: 0,474 ± 0,0423 unidade arbitrária versus grau 3: 0,580 ± 0,094 unidade arbitrária; p = 0,007).

**Conclusão:**

Os níveis plasmáticos de proteína 1 relacionada à uteroglobulina se associam com o grau da lesão pulmonar por inalação.

## INTRODUCTION

Burns are a major global health problem, with over 175,000 victims dying from these injuries annually.^(1)^ Most accidents are domestic and involve scalding liquids, fire, or contact with hot objects.^(1)^ Due to advances in care in recent years, mortality has decreased, and inhalation injuries are now the leading cause of death in burned patients.^(2)^ Significant morbidity and mortality are associated with lung injuries from inhaling smoke or the byproducts of combustion^(2)^ and can involve thermal injury, chemical irritation and systemic toxicity, or any combination of these. Lung injuries and skin burns increase the need for aggressive fluid resuscitation, which could lead to progressive pulmonary dysfunction, longer ventilation time, pneumonia or acute respiratory distress syndrome.^(3,4)^ Currently, however, there is no gold standard diagnostic strategy, and care is generally supportive.^(3,4)^

The consequences of smoke inhalation are often due to inflammatory responses that depend on a number of mediators, whose role is not yet clearly understood.^(3)^ Although mortality directly associated with smoke inhalation is relatively low (0 - 11%), it can be as high as 90% when combined with burns.^(5)^

The interaction between these mediators in the face of critical illnesses requires a more theoretical basis and clinical and laboratory research. In smoke inhalation patients, the pulmonary inflammatory response increases with each degree of inhalation injury, and weak pulmonary immune responsiveness is associated with mortality.^(6)^

Uteroglobin-related protein 1 (UGRP1), a secretoglobin, is a small protein in the CC16 family. Uteroglobin-related protein 1 is a 17 kD homodimeric protein specific to the lungs and is also known as secretoglobin SCGB3A2. Uteroglobin-related protein 1 appears to be highly specific to the airways and appears, similar to other pneumoproteins, to be a potential biomarker of lung damage. Uteroglobin-related protein 1 is an anti-inflammatory recombinant protein that has been shown to improve lung inflammation in animal models of lung injury,^(7-10)^ and probably in humans.^(11)^

To the best of our knowledge, UGRP1 has never been measured in burn lung injury. Thus, we hypothesized that UGRP1 levels could reflect disease severity and predict outcome in critically ill patients with inhalation burn injury.

On January 27, 2013, 235 people died and 143 others were severely injured due to a fire at a nightclub in Santa Maria, Rio Grande do Sul, which is in southern Brazil.^(9)^ A total of 88 patients were admitted to the intensive care unit (ICU) and diagnosed with severe smoke inhalation injury and varying degrees of burns. Eighteen patients were transferred from Santa Maria to the *Hospital de Clínicas de Porto Alegre*, Porto Alegre, Brazil, and 16 of them were included in this study.

This study investigated serum UGRP1 expression soon after burn and smoke inhalation injuries, aiming to correlate secretoglobulin with the severity of inhalation injury in critically ill patients.

## METHODS

A cohort of 18 critically ill victims of the Santa Maria fire disaster were transferred to the *Hospital de Clínicas de Porto Alegre*, a referral center 300km away from the scene. For 16 of the patients, informed consent to participate in the study was given by next of kin.

This study was approved by the Research Ethics Committee of the *Hospital de Clínicas de Porto Alegre* (Registry 13-0106).

We conducted a prospective observational study that included 16 patients admitted to the ICU of *Hospital de Clínicas de Porto Alegre* within 24 hours of injury. All patients were diagnosed with acute respiratory distress syndrome due to smoke inhalation and were on mechanical ventilatory support. Acute respiratory distress syndrome was defined according to the Berlin consensus.^(12)^ Flexible bronchoscopies (Olympus Medical System Corporation, Hamburg, Germany) were carried out in all patients.

Bronchoscopy criteria included carbonaceous sputum, soot in the nostrils, head or neck burns and smoke inhalation. Blood samples were collected at the time of ICU admission (mean time of 24 hours after inhalation injury). Data were collected on clinical variables and outcomes of interest, including age, sex, Sequential Organ Failure Assessment (SOFA) score,^(13)^ 24-hour fluid balance, body surface burned, hemodialysis, use of vasopressor agents, grade of inhalation injury, time spent on ventilation support, ICU length of stay, hospital length of stay, and 28-day and hospital mortality rates.

### Inhalation injury grading and sample processing

The degree of inhalation injury was determined by three experienced pulmonologists and classified into one of four categories (0, 1, 2 or 3) following Chou et al.: Grade 0 means no visible injury; Grade 1 involves mild edema or hyperemia, with or without soot; Grade 2 involves severe edema or hyperemia, with or without soot; and Grade 3 involves ulceration, necrosis and no cough reflex or bronchial secretion.^(6)^

Uteroglobin-related protein 1 plasma levels were determined in duplicate using an enzyme-linked immunosorbent assay (ELISA) with two monoclonal antibodies against human UGRP1, an antisurfactant, interleukin-6 (IL-6), interleukin-8 (IL-8), interleukin-10 (IL-10) and tumor necrosis factor (TNF) (ELISA Duo Set® and antibodies kit R&D Systems, Minneapolis, MN, USA). The values are expressed as arbitrary units/mL, and the results were compared with the degree of inhalation injury, which had been determined by bronchoscopy.

### Statistical analysis

Statistical analysis was conducted using IBM Statistical Package for Social Sciences (SPSS) Statistics 26.0 for Windows. Categorical data are described as absolute and relative frequencies. Numerical data are presented as the mean ± standard deviation. Univariable associations between numerical and categorial variables were tested using one-way analysis of variance. Pairwise comparisons were performed using Tukey’s HSD post hoc test. The association between UGRP1 plasma levels and inhalation injury severity adjusted for body surface burned was evaluated using analysis of covariance. Pairwise comparisons were performed using the least significant differences post hoc test. Marginal means are presented as the mean ± standard error. Univariable associations between other numerical variables were evaluated using Spearman’s ordinal correlation coefficient. Probability values < 0.05 were considered statistically significant.

## RESULTS

A total of 16 patients were prospectively included in the study, and their clinical characteristics and outcomes are presented in table 1. Table 2 shows the biochemical analyses. Mechanical ventilation was performed in volume-controlled mode with a Servo-s ventilator (Maquet, Rastatt, Germany). The median time spent on mechanical ventilation was 10 (2 - 23) days.

**Table 1:** Clinical characteristics and outcomes of the included patients

Variables	
Age (years)	23 ± 5
Male sex	11 (68)
Pneumothorax	5
Hemodialysis	3
Body surface burned > 20%	7
Shock (use of vasopressor)	11
Time on mechanical ventilation (days)	10 ± 6
ICU length of stay (days)	14 ± 9
Hospital length of stay (days)	19 ± 9
ICU mortality	1 (6.2)
28-day mortality	1 (6.2)
Hospital mortality	2 (12.5)
SOFA	
Day 1	4.5 ± 2.4
Day 3	4.7 ± 3.8
PaO_2_/FiO_2_	342 ± 139
24 hours fluid balance (L)	5733 ± 4917
Inhalation injury	
Grade 1	3 (18.7)
Grade 2	4 (25)
Grade 3	9 (56)

ICU - intensive care unit; SOFA - Sequential Organ Failure Assessment; PaO2/FiO2 - arterial partial

**Table 2:** Laboratory analyses of inhalation injury patients

Variables	
Peak lactate (mmol/L)	2.3 ± 1.9
Peak CRP (mg/dL)	190 ± 87
ACTH (pg/dL)	6.46 ± 5.21
TSH (mU/L)	1.42 ± 2.03
Free T4 (ng/dL)	21.17 ± 34.69
IL-10 (pg/mL)	31.38 ± 10.65
IL-6 (pg/mL)	16.28 ± 3.01
IL-8 (pg/mL)	54.06 ± 26.07
UGRP1 (OD/mL)	0.52 ± 0.11
Antisurfactant (OD/mL	0.43 ± 0.13

CRP - C-reactive protein; ACTH - adrenocorticotropic hormone; TSH - thyroid-stimulating hormone; IL - interleukin; UGRP1 - uteroglobin-related protein 1. Results are expressed as mean ± standard deviation.

**Figure 1:**
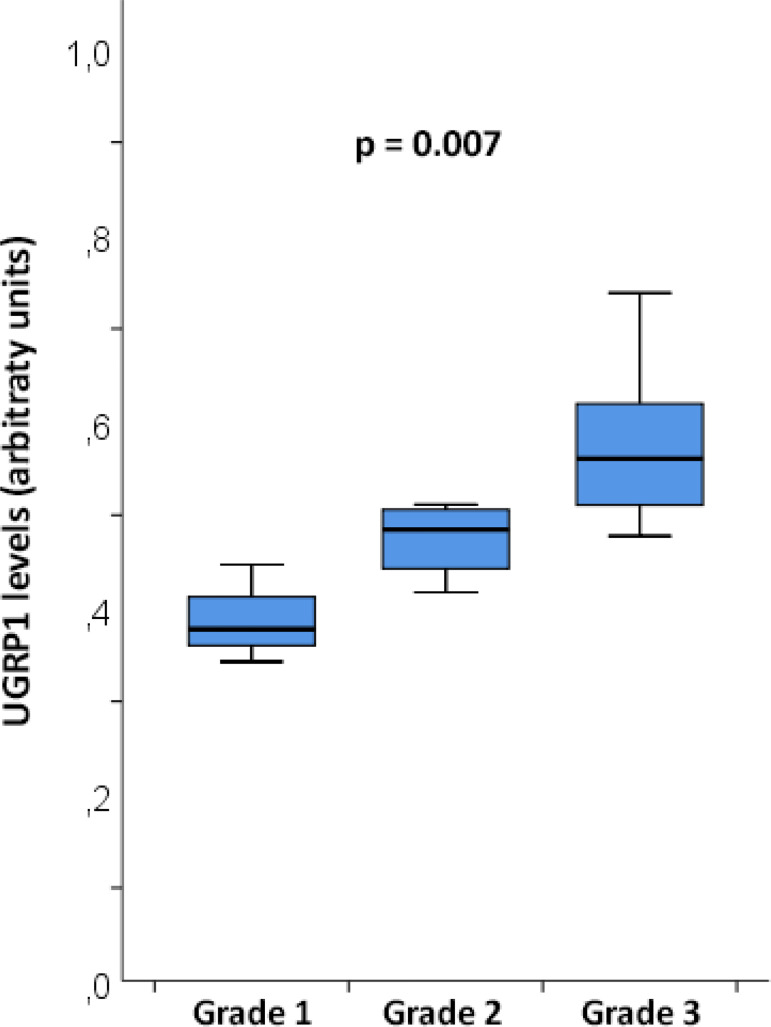
Association of uteroglobin-related protein 1 plasma concentrations and inhalation injury severity. UGRP1 - uteroglobin-related protein 1. p = 0.007.

Inhalation injury was classified as follows: three patients presented with Grade 1, four patients with Grade 2, and nine patients with Grade 3. Uteroglobin-related protein 1 plasma levels were related to inhalation injury severity (Grade 1: 0.39 ± 0.05 arbitrary units, Grade 2: 0.47 ± 0.04 arbitrary units, Grade 3: 0.58 ± 0.09 arbitrary units, p = 0.007) (Figure 1).

Post hoc analysis showed that Grade 3 injury was associated with higher levels of UGRP1 than Grade 1 injury (mean difference 0.19, p = 0.008). No statistically significant differences were observed between Grade 1 and Grade 2 injuries (p = 0.363) or between Grade 2 and Grade 3 injuries (p = 0.103).

**Figure 2:**
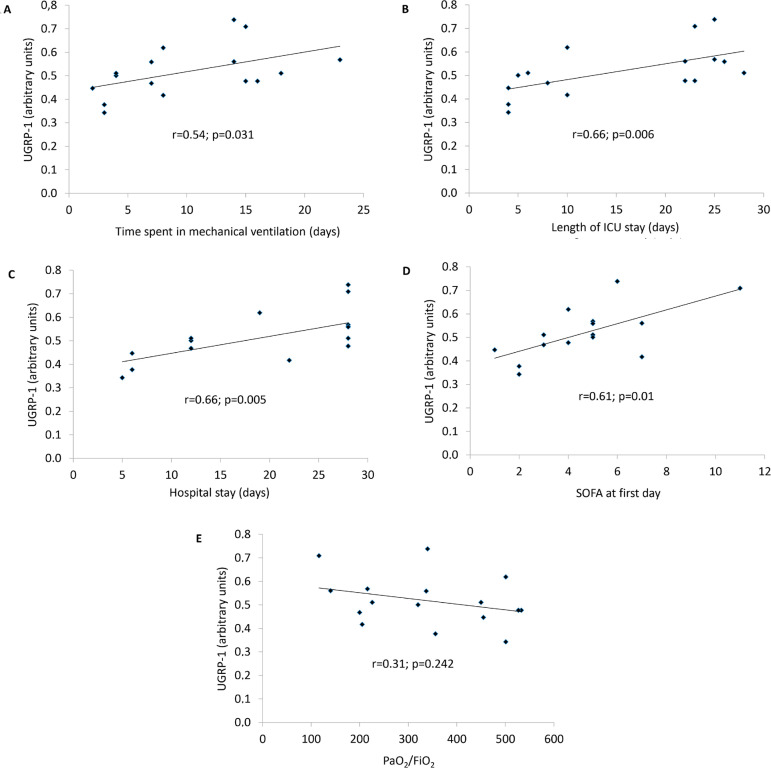
Correlation between uteroglobin-related protein 1 and time spent on mechanical ventilation (A), length of intensive care unit stay (B), length of hospital stay (C), Sequential Organ Failure Assessment at first day (D) and arterial partial pressure of oxygen/fraction of inspired oxygen (E). UGRP1 - uteroglobin-related protein 1; ICU - intensive care unit; SOFA - Sequential Organ Failure Assessment; PaO_2_/FiO_2_ - arterial partial pressure of oxygen/fraction of inspired oxygen.

A moderate positive correlation was observed between UGRP1 plasma levels and time spent on ventilation support (r = 0.54, p = 0.031); length of ICU stay (r = 0.66, p = 0.006); hospital stay (r = 0.66, p = 0.005); and SOFA score on the first day (r = 0.62, p = 0.01) but not between UGRP1 and the arterial partial pressure of oxygen/fraction of inspired oxygen (PaO_2_/FiO_2_) ratio on the first day (r = -0.31, p = 0.242). There was no significant association observed between UGRP1 and body surface burned (r = 0.46, p = 0.075) (Figure 2).

**Figure 3:**
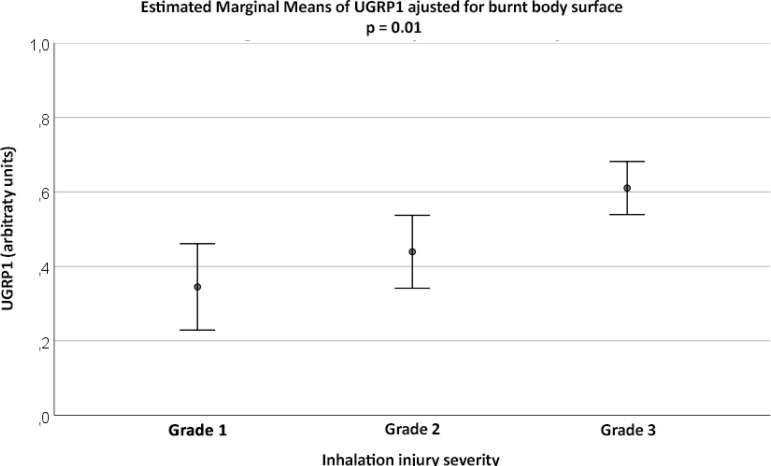
Uteroglobin-related protein 1 adjusted for body surface burned. UGRP1 - uteroglobin-related protein 1. p = 0.01.

The plasma levels of UGRP1 varied significantly according to the degree of airway injury even after adjustment for the body surface burned (p = 0.01) (Figure 3). Plasma levels of UGRP1 were significantly higher among patients with grade 3 injury (0.61 ± 0.03) than among those with grade 1 injury (0.35 ± 0.05; p = 0.003) and those with Grade 2 injury (0.44 ± 0.05; p = 0.02). There was no statistically significant difference in the plasma levels of UGRP1 among patients with grade 1 and 2 injuries (p = 0.132). It was observed that the body surface burned did not modify the association between UGRP1 and the degree of inhalation injury (p = 0.644). There was no statistically significant difference in IL-6 (grade 1 = 16.21 ± 4.17, grade 2 = 16.36 ± 2.85, grade 3 = 16.27 ± 3.10; p = 0.998), IL-8 (grade 1 = 42.93 ± 24.96, grade 2 = 60.17 ± 18.14, grade 3 = 55.04 ± 30.47; p = 0.707), IL-10 (grade 1 = 23.38 ± 11.87, grade 2 = 34.36 ± 5.65, grade 3 = 32.75 ± 11.61; p = 0.362), and tumor necrosis factor (TNF) (grade 1 = 6.19 ± 2.60, grade 2 = 5.25 ± 0.94, grade 3 = 4.81 ± 1.99; p = 0.572) for grade 1, 2 or 3 injury.

## DISCUSSION

The results of our study showed that UGRP1 plasma concentrations were associated with the severity of smoke inhalation injury. Uteroglobin-related protein 1 plasma concentrations were increased with each degree of inhalation injury.

Pro- and anti-inflammatory responses have been pointed out as potential determinants of organ dysfunction, such as acute respiratory distress syndrome,^(12)^ as well as of outcomes and survival. A Brazilian cohort suggested that the percentage of surface area burned is a more important risk factor for severity than inhalation injury.^(14)^ However, no specific mediator has been recommended for immunomodulation due to an incomplete understanding of both immunomodulation and the immune response to burns, trauma and sepsis. According to our results, UGRP1 concentrations indicate a systemic response to inhalation injury, since their levels were commensurate with the severity of the case. Moreover, we collected our plasma samples within 24 hours of injury, which minimized the effects of time on the concentration of immune mediators.

Our study suggests that UGRP1 is a determinant and/or marker of inhalation injury in burn patients. UGRP1, a pneumoprotein, is secreted in epithelial cells of the airway.^(7)^ The UGRP1 gene chromosomal region is associated with several allergic response genes. Although both UGRP1 and CC16 have similar amino acid sequences and tissue-specific expression, CC16 functions as an inflammatory agent, while UGRP1 is thought to act as an anti-inflammatory agent.^(15)^ Uteroglobin-related protein 1 could also be involved in the regeneration of lung epithelia.^(16)^ Currently, little information is available on the role of UGRP1 in the lungs. Thus, we propose that the increase in UGRP1 levels in the early phases after inhalation injury could be an adaptive mechanism to control lung damage.

There are several limitations to our study, the foremost of which is the small sample size. Second, it involves an inherent selection bias, since only severe respiratory failure patients suspected of inhalation injury were enrolled. Control plasma UGRP1 levels in normal volunteers were not determined. In addition, a dynamic measure of plasma levels could be of importance, i.e., the results would have been stronger if UGRP1 data were collected long after the inhalation injury, when lung conditions had improved. Finally, we provided no data elucidating the mechanism of UGRP1’s effects.

## CONCLUSION

In summary, we have demonstrated that uteroglobin-related protein 1, an anti-inflammatory lung secretoglobulin, is increased in human serum after smoke inhalation injury in critically ill burned patients and that uteroglobin-related protein 1 plasma levels are associated with inhalation injury severity.
